# The Effects of Different Wavelength UV Photofunctionalization on Micro-Arc Oxidized Titanium

**DOI:** 10.1371/journal.pone.0068086

**Published:** 2013-07-05

**Authors:** Yan Gao, Ying Liu, Lei Zhou, Zehong Guo, Mingdeng Rong, Xiangning Liu, Chunhua Lai, Xianglong Ding

**Affiliations:** 1 Center of Oral Implantology, Guangdong Provincial Stomatological Hospital, Southern Medical University, Guangzhou, China; 2 Department of Stomatology, Nanfang Hospital, and College of Stomatology, Southern Medical University, Guangzhou, China; 3 Department of Oral and Maxillofacial Surgery, Guangdong Provincial Stomatological Hospital, Southern Medical University, Guangzhou, China; 4 Department of Prosthodontics, Guangzhou Overseas Chinese Hospital, Jinan University, Guangzhou, China; University of Akron, United States of America

## Abstract

Many challenges exist in improving early osseointegration, one of the most critical factors in the long-term clinical success of dental implants. Recently, ultraviolet (UV) light-mediated photofunctionalization of titanium as a new potential surface treatment has aroused great interest. This study examines the bioactivity of titanium surfaces treated with UV light of different wavelengths and the underlying associated mechanism. Micro-arc oxidation (MAO) titanium samples were pretreated with UVA light (peak wavelength of 360 nm) or UVC light (peak wavelength of 250 nm) for up to 24 h. UVC treatment promoted the attachment, spread, proliferation and differentiation of MG-63 osteoblast-like cells on the titanium surface, as well as the capacity for apatite formation in simulated body fluid (SBF). These biological influences were not observed after UVA treatment, apart from a weaker effect on apatite formation. The enhanced bioactivity was substantially correlated with the amount of Ti-OH groups, which play an important role in improving the hydrophilicity, along with the removal of hydrocarbons on the titanium surface. Our results showed that both UVA and UVC irradiation altered the chemical properties of the titanium surface without sacrificing its excellent physical characteristics, suggesting that this technology has extensive potential applications and merits further investigation.

## Introduction

Pure titanium and titanium alloys are widely used as dental implants due to their excellent physiochemical properties and biocompatibility. The clinical long-term success of dental implants is related to their early osseointegration, thus the implant surface plays an important role in the progression [1], [2]. There are many reports on the effects of titanium surface treatment on the behavior of osteoblast-like cells, attempting to determine the optimum surface to promote early osseointegration. The modification of the surface at the micro-nanoscale level is generally considered to be more conducive to the attachment, spread and proliferation of osteoblast-like cells [3], [4]. Future trends in dental implant surfaces are concerned with biomimetic calcium phosphate coatings to enhance osteoconduction [5], [6].

Microarc oxidation (MAO) is a surface treatment that can produce a well-characterized, biocompatible titanium dioxide (TiO_2_) coating, incorporating calcium, phosphorus, and some well-distributed porous pits. The TiO_2_ layer provided by the MAO treatment has been shown to improve cellular response in vitro [7], [8] and promote de novo bone formation around the implant in vivo [9], [10]. These improvements were attributed to the incorporation of Ca and P and the porous morphology, which can increase mechanical interlocking between bone tissue and implant [11]. This interaction is termed “osteoconduction”, which is defined as appositional bone growth permitting bone formation on a surface or into pores [2]. The phenomenon of osteoconduction depends on the migration and attachment of osteogenic cells to the surface of the implant [12]. Attachment can occur when the cell itself directly adheres to the surface, which is the early stage of contact osteogenesis [13]. This phenomenon of contact osteogenesis is beneficial for early osseointegration [14], [15]. However, the bone-titanium contact percentage of current dental titanium implants is 50–75% at most [16], [17], and it drops to 49±12% after MAO treatment [9], [18]. Thus, the bioactivity of implant surfaces using MAO must still be improved for long-term success.

It is well known that TiO_2_ is an attractive semiconductor for certain photocatalytic applications, such as decontamination and bactericidal effects. Recently, the photofunctionalization of TiO_2_ has been studied as a method for implant surface treatment. These photofunctionalized titanium surfaces display substantially enhanced osteoconductivity and improved early osseointegration capabilities, which can be attributed to chemical alterations within the TiO_2_ coating [19], [20]. The phenomenon of superhydrophilicity of TiO_2_ was discovered in 1997 [21]; this is an obvious photochemical reaction. Ultraviolet (UV) treatment of the age-related degradation (for 4 weeks) of a titanium surface also increases the bioactivity over the freshly prepared surface in terms of protein adsorption, cellular attachment and proliferation [22], [23]. However, the mechanism of the generation of superhydrophilicity is still unknown. Currently, two mechanisms have been proposed [20], [22]: one is the generation of surface vacancies at bridging sites, resulting in the conversion of Ti^4+^ to Ti^3+^, which is favorable for dissociative water adsorption to form basic Ti-OH groups. The other is the elimination of hydrocarbons on the TiO_2_ coating. UV light energy greater than 3.2 eV can induce TiO_2_ photocatalysis, which corresponds to UVA and UVC light of approximately 360 nm and 250 nm, respectively [23]–[25]. In the majority of reports, UVC irradiation was used and regarded as a new surface treatment to improve the hydrophilicity and enhance the protein adsorption and cell response [20], [24], subsequently forming de novo bone rapidly around the implant [20]. There is some disagreement in reports using UVA illumination on the probable causes of the different photocatalytic effects on different titanium surfaces. UV-induced photofunctionalization is effective on various titanium surfaces; however, there are currently so many different implant surfaces, it is difficult to evaluate which surface exhibits a better photocatalytic effect.

Our previous study showed that MAO treatment is capable of creating a micro-nano hybrid titanium surface [10]. Excitingly, a recent report suggested that UVC photofunctionalization and micro-nano hybrid topography combine for a synergistic effect on the biological properties of titanium [26]. Another study further showed that the micro-nano hybrid surface has the ability to alleviate biological aging of UVC-photofunctionalized titanium [27]. A subsequent question to be answered in this study is whether the MAO surface exhibits superior biological activity after UVC treatment. An additional purpose of this study is to compare the influence of different wavelengths of UV irradiation to find out which type of UV light better enhances the osteoconductivity of the MAO surface. The protein and cell behaviors on the MAO surface, the dependence of the behavior on the type of UV treatment, and the possible mechanism responsible for this behavior are also examined.

## Materials and Methods

### Cell Line

The cell line (human osteoblast-like cells, MG-63) used in the present study was provided by the Laboratory of cell biology of Southern Medical University, Guangzhou, China. Reference: Badique F, Stamov DR, Davidson PM, Veuillet M, Reiter G, et al. (2013) Directing nuclear deformation on micropillared surfaces by substrate geometry and cytoskeleton organization. Biomaterials 2013 34:2991–3001.

### Titanium Sample Preparation

The titanium disks (15 mm in diameter, 1 mm in thickness) were cut from commercial titanium rods. The titanium surface was ground with 400 grit, 600 grit, 800 grit and 1000 grit SiC papers, and then ultrasonically cleaned with acetone, absolute ethanol and distilled water for 15 min in series. In accordance with our previous work [10], the titanium disks were treated by MAO in an aqueous electrolyte solution containing 3.5% glycerophosphate disodium salt pentahydrate (C_3_H_7_Na_2_O_6_P·5H_2_O) and 1.2% calcium acetate monohydrate ([CH_3_COO]_2_Ca·H_2_O) (voltage 350 V, frequency 800 Hz) for 30 seconds, and then ultrasonically rinsed with distilled water for 15 min. Among all the MAO coated disks, 1/3 of the samples were treated with a 15 W UVA mercury lamp, which generates maximum intensity light at 360 nm. Another 1/3 of the samples were treated with a 15 W UVC bactericidal lamp, which generates maximum intensity light at 250 nm. These samples were treated with the UV light under ambient conditions for 24 h.

### Surface Analysis of the Samples

The surface morphology was observed using a scanning electron microscope (FESEM; S-3700N, Hitachi, Japan) with an energy dispersive X-ray spectrometer (EDXS; ESCALAB 250, Thermo Fisher Scientific, USA). The hydrophilicity of the titanium surface was measured by the contact angle of 1 µl H_2_O using a contact angle measuring device (OCA15; Dataphysics, Germany). The crystalline structure of the titanium surface was examined by X-ray diffraction (XRD) (D8 ADVANCE; Bruker, Germany). A three-dimensional profile of the titanium surface was observed with an optical profilometer (Breitmeier Messtechnik GmbH; BMT, Germany). The surface roughness parameter Ra was quantified over a scanning area of 300 µm×300 µm. The elemental composition of the titanium surface was measured by X-ray photoelectron spectroscopy (XPS) (ESCALAB 250; Thermo Fisher Scientific, US). Photoelectrons generated by monochromatic Al Kα X-ray radiation at 150 W (15 kV, 10 mA) were analyzed with a hemispherical electron energy analyzer. Survey scans and higher-resolution narrow scans of the main characteristic peaks (Ti 2p, C 1s, O 1s, Ca 2p and P 2p) were recorded at a take-off angle of 90°. The binding energy was corrected by the C 1s (hydrocarbons C-C, C-H) contribution at 284.8 eV. Casaxps software was used for the semi-quantitative analysis of the surface chemical composition.

### Bioactivity Evaluation Assay

Each group of samples was immersed in a plastic vial containing 35 ml of SBF (M & C Gene Technology, China) and kept at 37°C for one week and three weeks to evaluate their bioactivity. After the samples were removed from the SBF solution, they were washed with distilled water and air dried. The surface morphology of each sample was observed by SEM. The crystalline component of the titanium surface was examined by XRD to evaluate the apatite-forming ability.

### Protein Adsorption Assay

Bovine serum albumin (BSA) was used as a model protein. 300 µl of protein solution (1 mg/ml protein/distilled water) was pipetted onto each titanium disk in a 24-well plate. After different periods of incubation (2 h, 6 h or 24 h) at 37°C, the non-adherent protein solution was removed. 10 µl each of the initial and removed solutions were mixed with micro bicinchoninic acid at 37°C for 30 min. The amount of protein was quantified by a microplate reader (SpectraMax M5, Molecular Devices, USA) at 562 nm.

### Osteoblastic Cell Culture

Human osteoblast-like MG-63 cells, derived from a human osteosarcoma, were cultured in Dulbecco’s modified Eagle’s medium (DMEM; HyClone, Thermo Fisher Scientific, US) containing 10% fetal bovine serum (FBS) and a 1% antibiotic mixture (penicillin-streptomycin; HyClone) at 37°C under a humidified atmosphere of 5% CO_2_. The MG-63 cells were trypsinized by 0.25% trypsin-EDTA (HyClone) when they reached 80% confluence, and were then seeded onto the testing disks in 24-well plates at a density of 1×10^4^ cells/cm^2^. The culture medium was renewed every two days.

### Cell Attachment Assay

Initial cell attachment was evaluated by calculating the fluorescent-stained nuclei on the tested disks after 1 h, 2 h and 4 h of incubation. After each selected period of time, the samples were rinsed with phosphate buffered saline (PBS, HyClone) and fixed with 4% parafomaldehyde for 30 min, then immersed in 0.1% Triton X-100 in PBS for 5 min, and blocked with 1% bovine serum albumin (BSA/PBS) for 30 min at room temperature. The adherent cells were stained with Hoechst 33342 (Sigma-Aldrich, USA) for 5 min in dark ambient conditions. The amount of cells attached onto the disks was evaluated by counting the number of stained nuclei using fluorescence microscope (IX51, Olympus, Japan) images at a magnification of 100× over an area 1800 µm×1350 µm. On three disks of each group, four different fields of each sample were randomly selected for counting using Image J version 1.42q analyzing software (National Institutes of Health, Bethesda, MD).

### Cell Morphology

After 1 h and 4 h of culturing, the cells were fixed with 2.5% glutaraldehyde for 2 h, dehydrated with 30%, 50%, 70%, 90% and absolute ethanol in series, and then tertiary butyl alcohol three times, and then freeze-dried. Each sample was sputter-coated with Au/Pd for observation using SEM (JSM-6330F, JEOL, Japan) at a magnification of 3000×. The cytoskeletal arrangements were observed by immunofluorescence staining. After 24 h of incubation, the disks were rinsed with PBS and fixed with 4% parafomaldehyde for 30 min, immersed in 0.1% Triton X-100 in PBS for 5 min, and blocked with 1% bovine serum albumin (BSA/PBS) for 30 min at room temperature. Protected from light, the adherent cells were then stained with a phalloidin/PBS mixture (1/40 v/v) (Alexa Fluor 635, Invitrogen, USA) for 30 min, followed by incubation with PBS containing 10 µg/ml Hoechst 33342 for 5 min. The specimens were stored at low temperature in the dark and then observed by a fluorescence microscope at a magnification of 400×.

### Cell Proliferation Assay

MG-63 cells were seeded onto each sample in 24-well plates at a density of 1×10^4^ cells/cm^2^. After 1 d, 3 d and 5 d of incubation in a humidified atmosphere of 5% CO_2_ at 37°C, the specimens were rinsed three times with PBS and then transferred into a new 24-well plate. 500 µl DMEM was added to each well, followed by 100 µl methyl tetrazole sulfate (MTS) (CellTiter 96® Aq_ueous_ Non-Radioactive Cell Proliferation Assay, Promega, USA). After each prescribed time period, 100 µl of the culture solution was transferred to a 96-well plate and measured by a microplate reader (SpectraMax M5, Molecular Devices, USA) at 490 nm.

### Alkaline Phosphatase (ALP) Activity

MG-63 cells were seeded onto each sample surface at the same density as in the cell proliferation assay. After 3 d, 7 d and 14 d of incubation, the cultured cells were washed three times with PBS solution and incubated with 200 µl 1% TritonX-100 for 40 min at 37°C. For colorimetry, the cells were then incubated in ALP reagent SIGMA FAST *p*-nitrophenyl phosphate (*p*-NPP) (Sigma-Aldrich, Missouri, USA) for 30 min at 37°C. The ALP activity was measured by the amount of *p*-NP formed, as determined by a microplate reader (SpectraMax M5, Molecular Devices, USA) at 405 nm.

### Statistical Analysis

The experimental data were expressed as mean±standard deviation (SD). Statistical analysis was performed by SPSS 13.0 software (SPSS Inc., Chicago, USA). One-way ANOVA was used to assess the effects of the different wavelength UV treatments. If necessary, the post-hoc Bonferroni test was used in the case of homogeneity of variance. Dunnett’s T3 test was used in the case of heterogeneity of variance. *p*<0.05 was considered statistically significant.

## Results

### Surface Morphology and Elemental Composition of the Specimens

**Figure 1:**
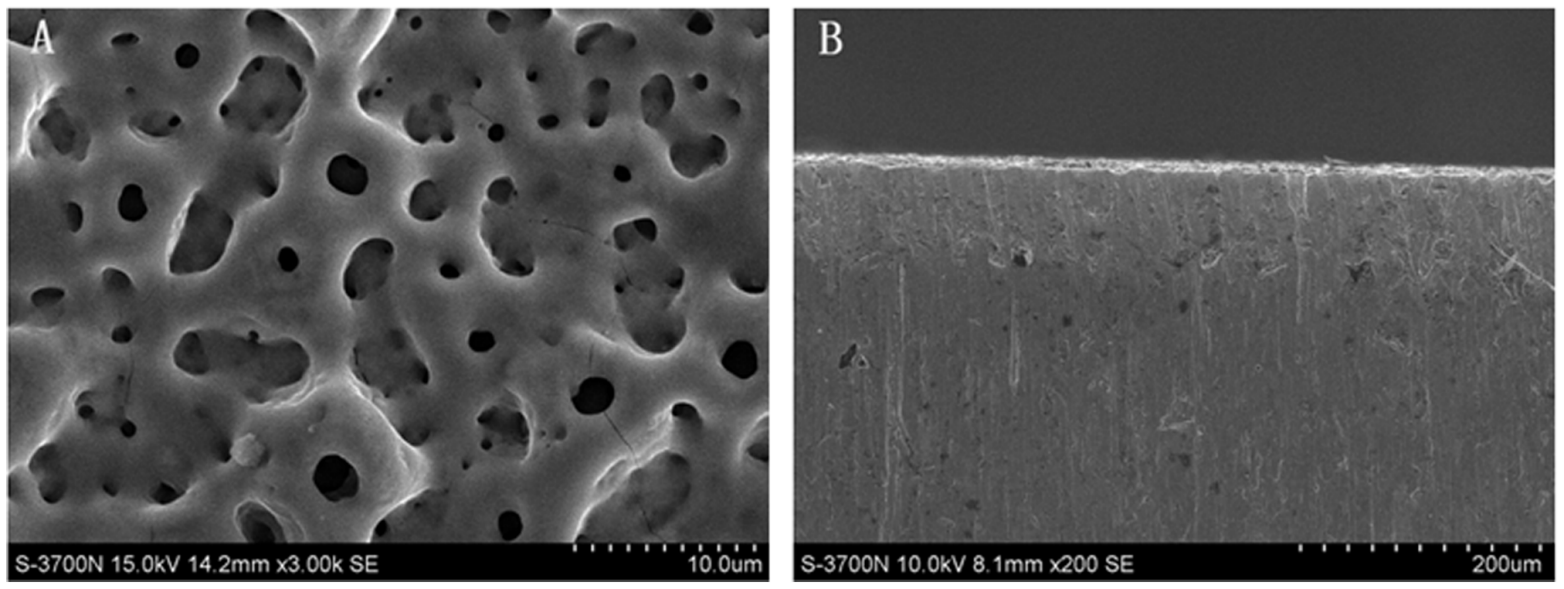
SEM images of the titanium surface used in this study. (A) MAO titanium surface (3000X) and (B) cross-sectional view (200X).

FESEM showed that the UV treatments did not significantly change the topography of the MAO coating. All the coating surfaces showed abundant pores with diameters varying from 1–10 µm. These pores were well separated and distributed, and the matrix appears quite dense; some nanoscale pores were also observed (Fig. 1A). The cross-sectional image is shown in Fig. 1B, and the thickness of the oxide film was approximately 10 µm. The surface elemental composition consisted primarily of Ti, O, Ca, P and C, as detected by EDX.

### Surface Roughness and the Crystalline Component of the Specimens

The surface roughness parameters Ra of the MAO, UVA-treated and UVC-treated surfaces were 1.262±0.053 µm, 1.288±0.072 µm and 1.237±0.045 µm, respectively, which shows that neither UVA treatment nor UVC treatment changes the surface roughness. Both UVA and UVC irradiation do not alter the crystalline component of the MAO surface. The coatings of all the specimens are composed of anatase (JCPDS # 21-1272) and rutile (JCPDS # 21-1276), and the Ti (JCPDS # 44-1294) peaks are attributed to the substrate.

### Changes in the Hydrophilicity of the Surface

**Figure 2:**
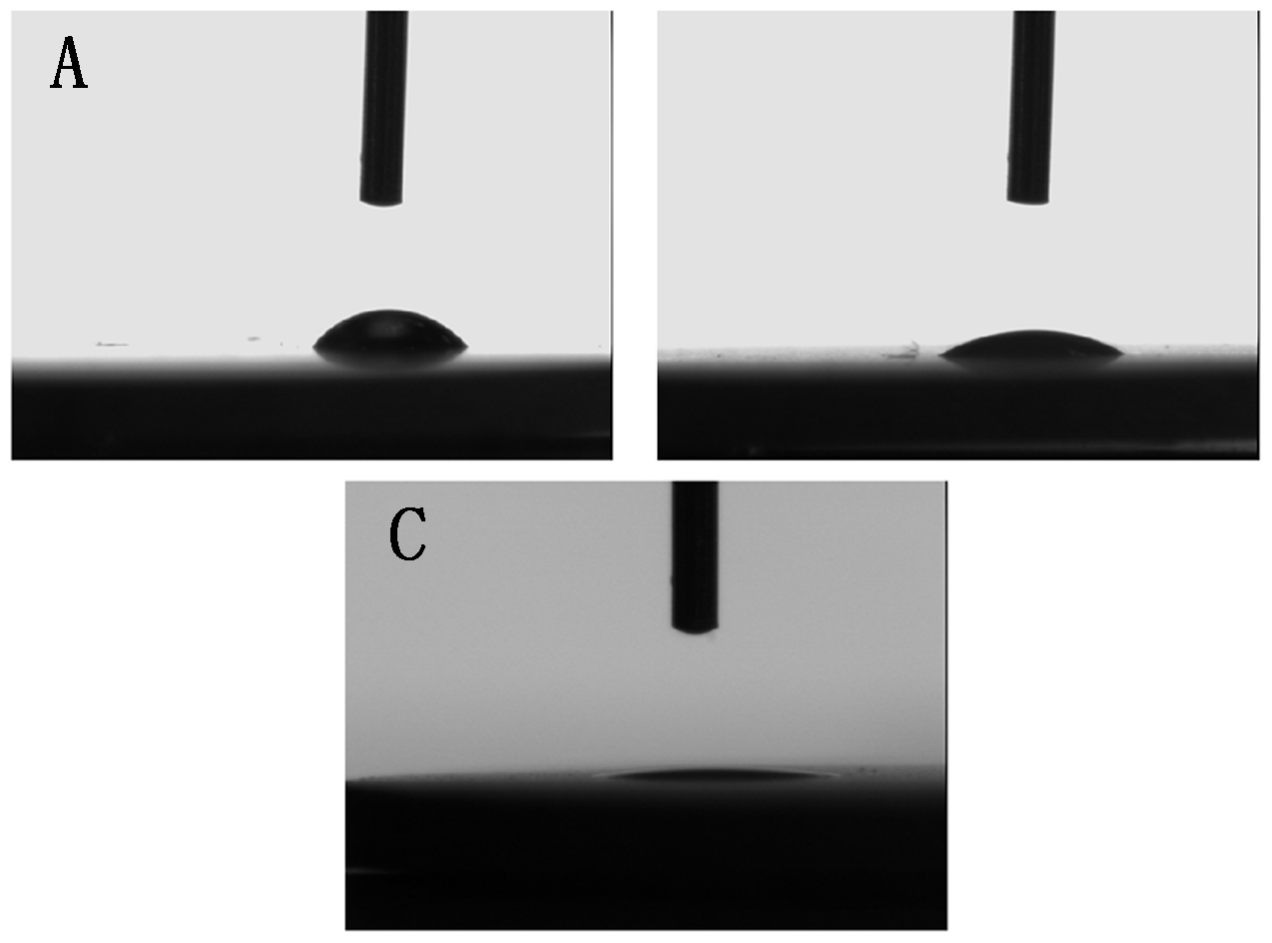
Photographic images of H_2_O droplets pipetted onto the titanium surfaces. (A) MAO, (B) UVA-treated and (C) UVC-treated.

The contact angles of a water droplet on the MAO, UVA-treated and UVC-treated surfaces (Fig. 2) were 65.34°, 44.64° and 3.41°, respectively, indicating that the hydrophilicity of the MAO surface was affected by both UVA and UVC treatment.

### Surface Chemical Species of the Specimens

According to the XPS measurements, the coatings of the three samples are composed of Ti, O, C, Ca, and P. These results agree with those obtained by EDX; a small amount of N on the surface is also observed, possibly due to contamination from the air.

**Figure 3:**
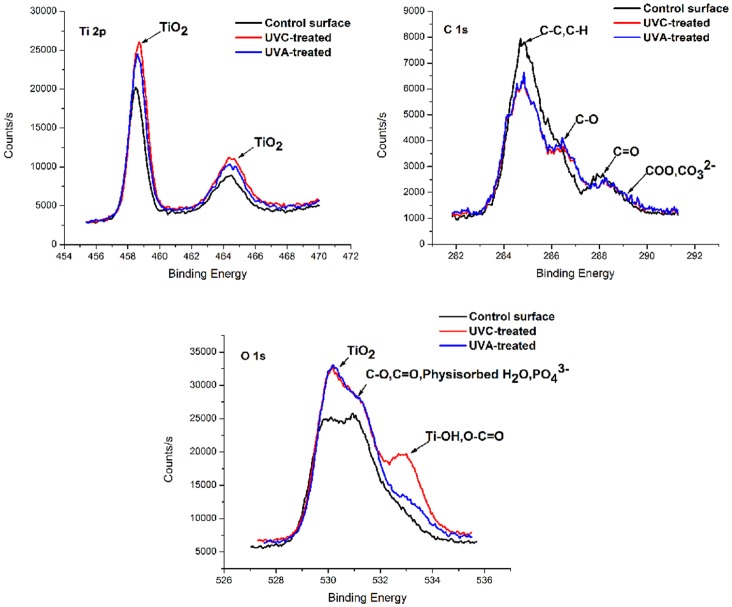
XPS high-resolution spectra of the titanium surfaces after UV treatment. (Ti 2p, C 1s and O 1s).

Looking at the XPS high-resolution spectra, Ca 2p and P 2p show no obvious change after UVA or UVC irradiation. The Ti 2p, O 1s and C 1s spectra of the three samples are shown in Fig. 3. Binding energies typical for Ti^4+^ [28], [29] were detected in the Ti 2p spectra at 458.5 eV and 464.2 eV, corresponding to Ti 2p3/2 and Ti 2p1/2, respectively. The Ti 2p peaks were shifted to a higher energy for the UV-treated surfaces compared with the MAO surface, especially for the UVC-treated surface. According to the literature[29]–[31], four peaks are fitted to the C 1s spectra. The predominant peak at 284.8 eV is attributed to hydrocarbons (C-C, C-H), and the other three peaks at 286.4 eV, 288.0 eV and 288.9 eV represent C-O, C = O and O-C = O, respectively. The XPS spectra revealed that the C 1s peak at 284.8 eV was markedly diminished after UVA or UVC irradiation, suggesting that both wavelengths of light could reduce the amount of hydrocarbons adsorbed on the MAO surface. Three peaks are assigned to the O 1s spectra according to the literature [30], [31]. The predominant peak at 530.1 eV is attributed to O 1s in TiO_2_ (O^2−^), and the peak at 531.3 eV corresponds to O 1s in PO_4_^3−^ [32], C-O, C = O [30], [31] and physisorbed H_2_O [33]. The third peak at 532.8 eV is assigned to O 1s in Ti-OH [34] and O-C = O [30], [31]. The XPS spectra showed increases in the three peaks of O 1s after both UVA and UVC treatments; the increase in the peak intensity at 532.8 eV was more pronounced for the UVC-treated surface than for the UVA-treated surface. The changes seen in the peaks at 288.9 eV in the C 1s spectra and at 532.8 eV in the O 1s spectra suggest that the increase of the O 1s peak at 532.8 eV is due to the generation of Ti-OH on the coating after UV treatment, especially on the UVC-treated surface.

### Apatite-forming Ability under Different Wavelengths of UV Irradiation

**Figure 4:**
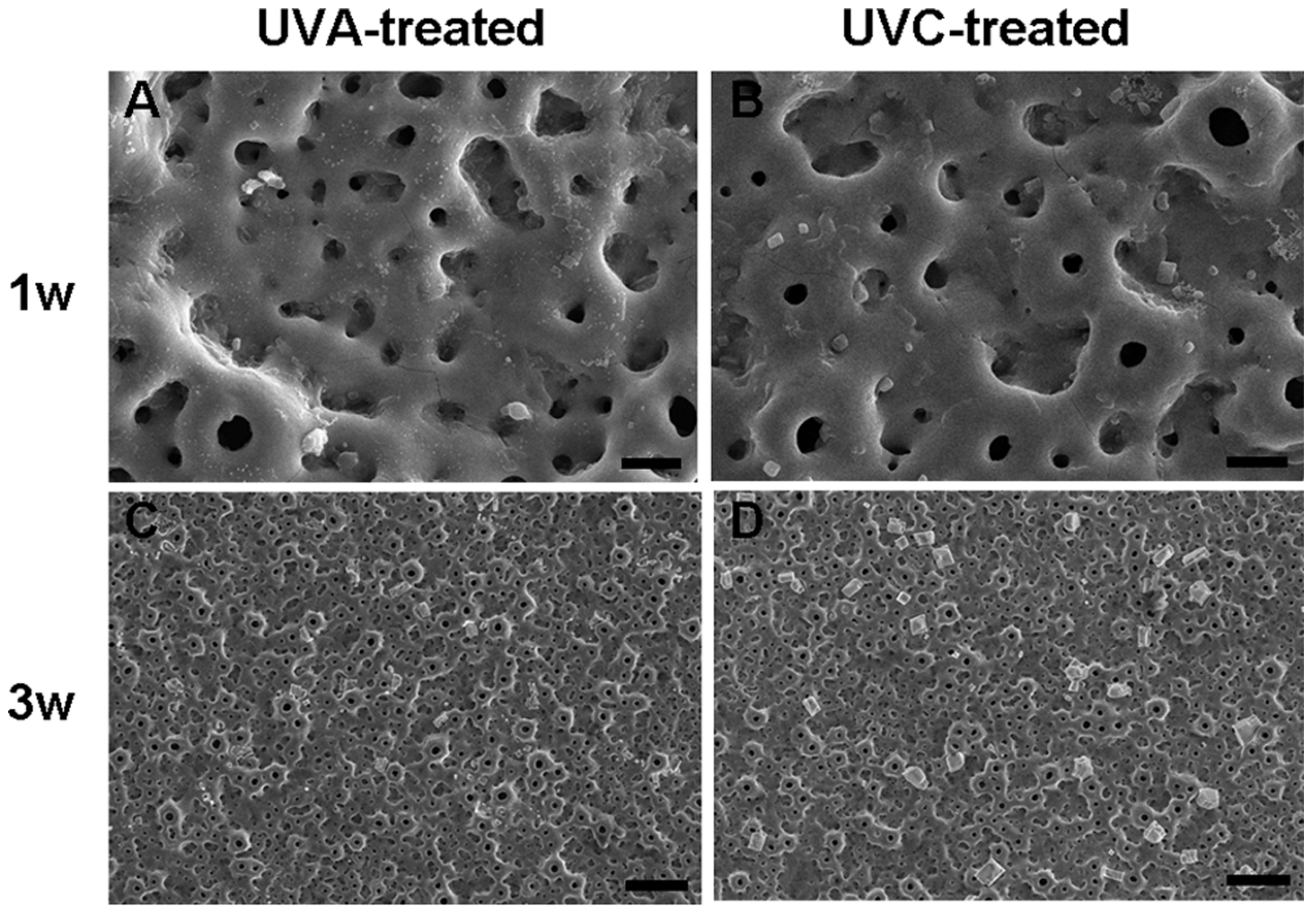
SEM images of the titanium surfaces after immersion in SBF. SEM micrographs of (A, C) UVA-treated and (B, D) UVC-treated surfaces after immersion in SBF for (A, B) one week (3000X, bar = 5 µm) and (C, D) three weeks (500X, bar = 20 µm).

The SEM images showed no obvious apatite formation on the MAO coating after immersion in SBF for three weeks. This result is in good agreement with previous research [19], which reported that apatite formation is difficult to induce on a pure MAO surface. After immersion of the UV-treated samples in SBF for one week, a small amount of granulated precipitate was seen on the UVA-treated coating (Fig. 4A), and many small blocks were deposited on the UVC-treated surface (Fig. 4B). As shown in Fig. 4C and Fig. 4D, the small blocks on the surface grew bigger after immersion for three weeks, especially on the UVC-treated surface.

**Figure 5:**
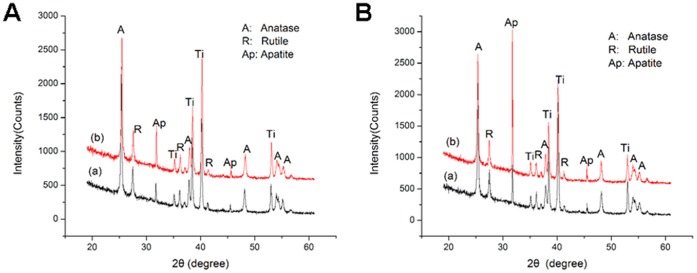
XRD spectra of the titanium surfaces after immersion in SBF. (A) one week and (B) three weeks. (a) UVA-treated and (b) UVC-treated.

Fig. 5A and Fig. 5B shows the XRD spectra of the UV-treated surfaces after immersion in SBF for one week and three weeks. New XRD peaks appeared, which were attributed to apatite (JCPDS # 09-0432). The strongest apatite peak appears at 31.8°, corresponding to the (211) diffraction plane of the standard XRD pattern of hydroxypatite (HA) (JCPDS # 09-0432) [35]. The intensity of the apatite peaks enhances with increasing immersion time for both UV-treated samples, and particularly for the UVC-treated coating (Fig. 5B (b)). In combination with the SEM images shown in Fig. 4, we can conclude that the growth of apatite on the UVC-treated surface is faster than on the UVA-treated surface.

### Enhanced Rate of Protein Adsorption and Cell Attachment after UVC Irradiation

**Figure 6:**
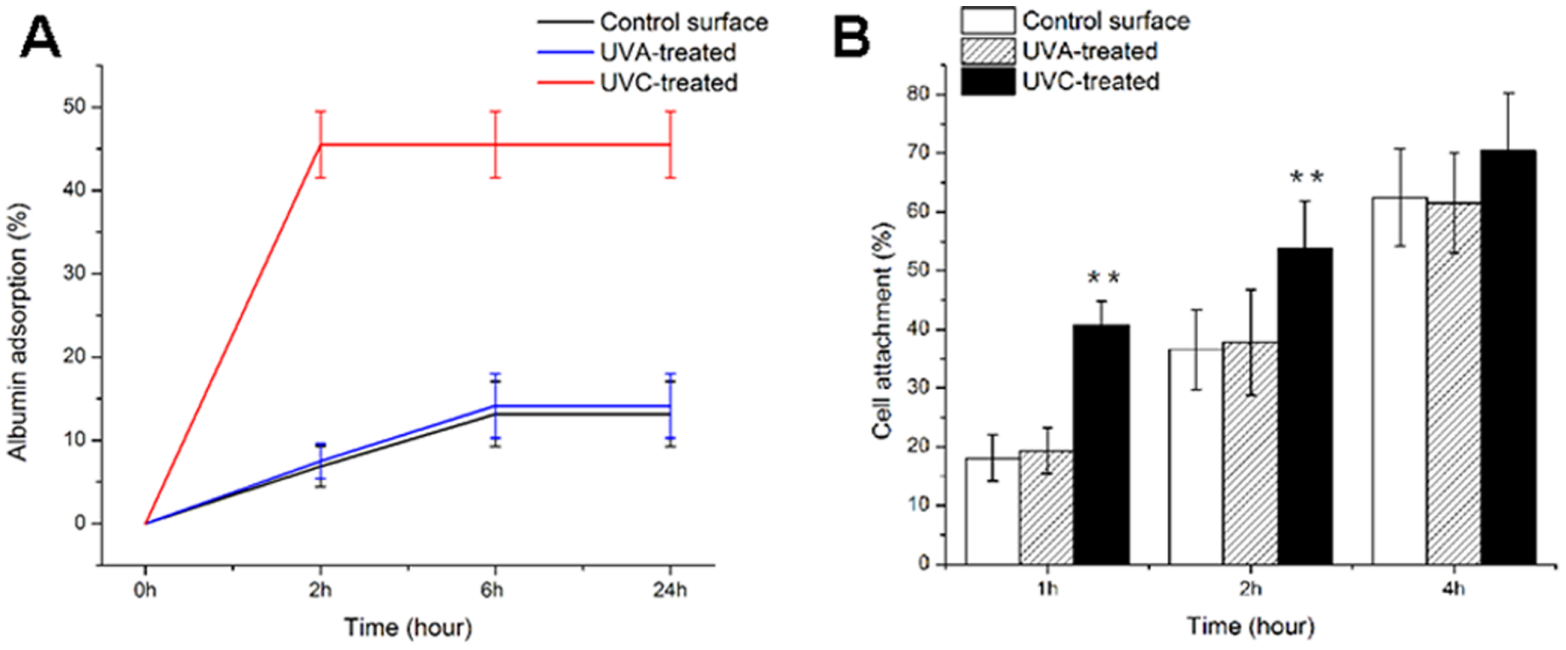
Albumin adsorption and cell attachment to the surfaces. (A) The rate of albumin adsorption to the titanium surface after incubation for 2 h, 6 h and 24 h. (B) The rate of osteoblasts attached to the titanium surfaces after 1 h, 2 h and 4 h incubation (mean±SD, n = 6; **p<0.01, *p<0.05).

Of the two different wavelengths of UV light, the UVC treatment promoted the adsorption of albumin on the MAO coating. As shown in Fig. 6A, the albumin adsorption rate on the UVC-treated surface peaked after a 2 h incubation. The UVA-treated surface displayed no significant difference in the protein adsorption compared with the MAO surface.

Initial cell attachment was measured by counting the fluorescent-stained nuclei. As shown in Fig. 6B, the rate of cell attachment increased with increasing incubation time (1 h, 2 h and 4 h) and was more than 60% on the different surfaces after 4 h of incubation. The UVC-treated coating exhibited better performance than the other two coatings, especially after 1 h and 2 h of incubation (*p*<0.01), indicating that UVC treatment accelerates cell adhesion. No statistical difference was found between the MAO and UVA-treated surfaces.

### Cellular Spread and Cytoskeletal Development on the Different Surfaces

**Figure 7:**
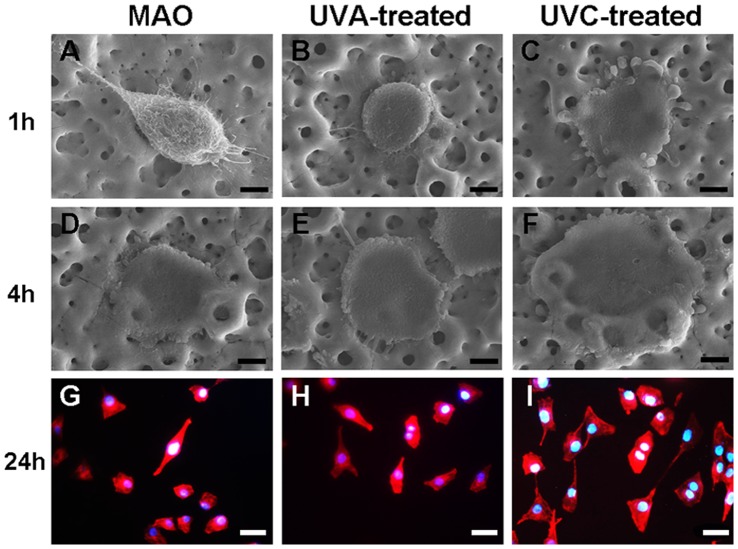
Initial morphologies of the MG-63 osteoblasts on the titanium surface. (3000X, bar = 10 µm) SEM images of cells on the MAO, UVA-treated and UVC-treated surfaces after (A–C) 1 h and (D–F) 4 h incubation; (400X, bar = 50 µm) Fluorescence microscopy images of cells on the MAO, UVA-treated and UVC-treated surfaces after (G–I) 24 h incubation.

The morphology of the MG-63 cells cultured on the different surfaces for 1 h and 4 h are shown in Fig. 7A–F. After 1 h of incubation, the cells on the MAO coating were oval-shaped, and did not show complete contact with the surface. Cells on the UVA-treated surface were round and showed some slender filopodia, while numerous sturdy lamellipodia were present on the UVC-treated surface. After 4 h of incubation, the cells on the the UV-treated surfaces stretched further, especially on the UVC-treated surface.

Fig. 7G–I shows the fluorescence microscopy images of the cells after being seeded onto different surfaces and incubated for 24 h. The majority of the cells displayed a similar morphology – that of a triangle or polygon. However, the cells on the MAO and UVA-treated surfaces could have spread further, while the cellular cytoskeleton on the UVC-treated surface exhibited a more extensive arrangement and formed actin networks, indicating that communication among cells began to be established.

### Enhanced Cell Proliferation and Differentiation after UVC Irradiation

**Figure 8:**
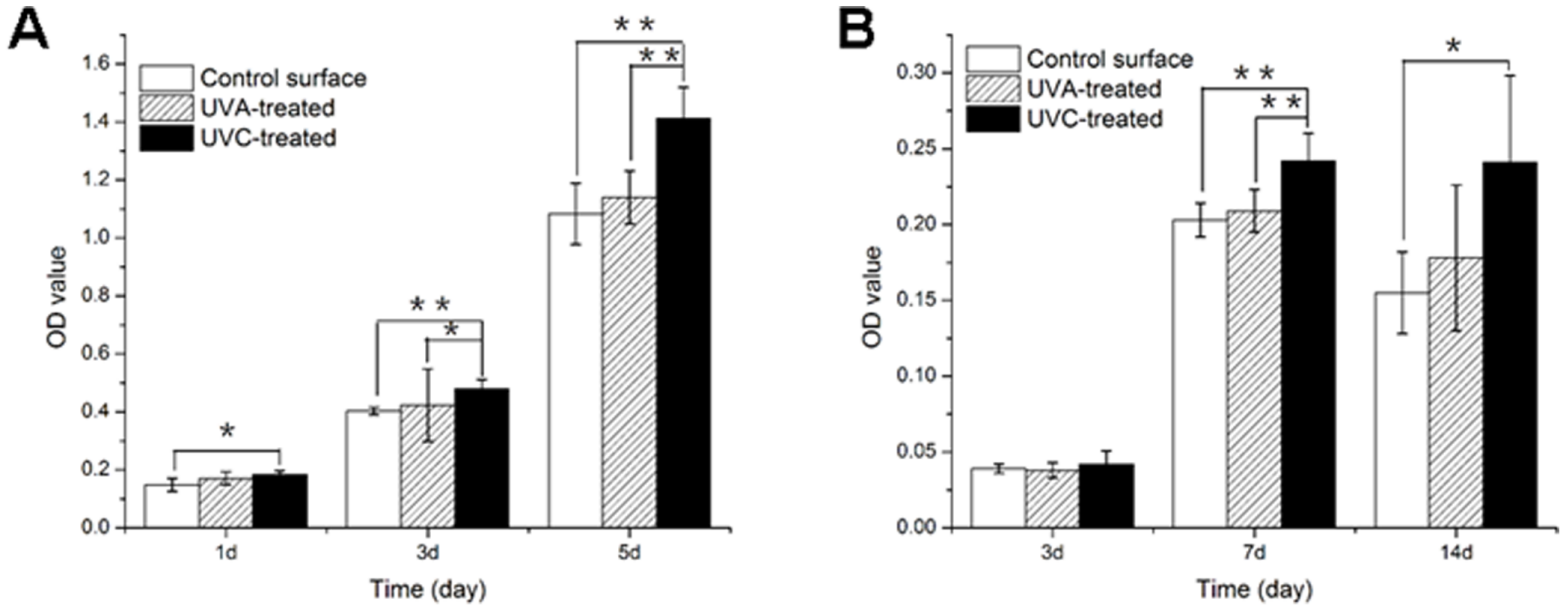
Proliferation and alkaline phosphatase (ALP) activity of osteoblasts. (A) Osteoblast proliferation on the titanium surfaces after incubation for 1 d, 3 d and 5 d. (B) Alkaline phosphatase activity of osteoblasts on the titanium surfaces after incubation for 3 d, 7 d and 14 d (mean±SD, n = 6; **p<0.01, *p<0.05).

The cell proliferation was evaluated by an MTS assay after 1 d, 3 d and 5 d of incubation. As shown in Fig. 8A, the cell proliferation increased over time on the different surfaces. At one day, there were more cells adhered to the UVC-treated surface than to the MAO surface (*p*<0.05). After 3 d and 5 d, the UVC-treated surface showed better performance than the other two surfaces (p<0.05), particularly after five days, when a significantly higher proliferative activity was observed (*p*<0.01).

The ALP activity was measured to evaluate the early cell differentiation on the different surfaces. As shown in Fig. 8B, the ALP activity on day seven was significantly higher than on day three on the different surfaces. However, after 14 d of incubation, the ALP activity of each surface decreased to varying degrees. Compared to the other two surfaces, the ALP activity of the UVC-treated surface displayed superior performance, especially on day seven (*p*<0.01).

## Discussion

This study demonstrates that UVC treatment can be used to substantially improve the titanium-mediated enhancement of osteoconductivity. The same effect was not observed on titanium surfaces that were treated with UVA light. The number of MG-63 cells on the UVC-treated surface increased by more than two-fold after a 1 h incubation period. Significant differences in the spreading of the cells were also observed: the spreading behavior of the MG-63 cells was delayed on the MAO surface, whereas it was expedited on the UVC-treated surface. The enhanced biological activity also resulted in increased osteoblastic proliferation and differentiation, as shown by the ALP activity assay. It is known that the initial biological response of a titanium surface in vivo is the adsorption of protein. In terms of the early albumin adsorption rate, the amount of protein adsorbed on the control group titanium surface was less than 10% after a 2 h incubation. The protein adsorbed on the UVC-treated surface was seen to increase by more than five-fold. Protein adsorption and cell attachment are key biological steps in the successful establishment of early osseointegration [14], [20]. However, based on investigations at the molecular level, it is unclear whether there exists a causal relationship between them. Most reports suppose that integrins, which are cell surface receptors, play the role of mediator between cells and proteins adsorbed on the material surface [2], [36]. Some adherent proteins, such as albumin, serve as carriers of important molecules to cells, for example, hormones and calcium [22]. The present study demonstrated that UVC-treated surfaces enable an increase in the albumin adsorption. Consequently, this increased protein adsorption may result in increased attachment of cells because of the interaction between proteins and cells.

The chemical alteration of a titanium surface plays a crucial role in its hydrophilicity, protein adsorption and cell attachment. To determine what chemical changes occurred as a result of the UV treatment, the MAO coating surfaces were carefully characterized in the present study. The XPS spectra show that the predominant peak at 284.8 eV, ascribed to oxygen-containing hydrocarbons adsorbed on the MAO surface, decreased after UVA and UVC irradiation, whereas the peak at 532.8 eV, attributed to basic Ti-OH, increased. Interestingly, the effect of both UV treatments on the hydrocarbons was similar, while the effect on the Ti-OH content differed significantly depending on the wavelength of the UV treatment. Although both UVA and UVC treatment decreased the amount of hydrocarbons on the MAO coating surface, the underlying mechanisms are different: the UVA-mediated photocatalytic reaction of TiO_2_ can remove hydrocarbons on the MAO coating surface, while UVC light directly decomposes hydrocarbons [24], [37]. Photolytic and photocatalytic degradation are two different concepts. They can be achieved by direct reaction with photons and oxygen vacancies, and/or by indirect reaction with OH radicals during UV irradiation [38]. The formation of Ti-OH on a titanium surface under UV irradiation is attributed to the conversion of Ti^4+^ to Ti^3+^ and the generation of oxygen vacancies, which are able to react with absorbed water [18]. Contrary to UVC illumination, UVA light does not efficiently produce OH radicals which are the key factors in photolytic reaction; this may be attributed to the lower light intensity [38]. Therefore, the removal of hydrocarbons by UVA light is expected to be achieved by photocatalytic degradation [38], [39]. However, it should be noted that the two proposed mechanisms of enhanced hydrophilicity [20], [22] should not be considered independent of each other. More importantly, our results show that the different degrees of hydrophilicity generated after UV irradiation contribute to the amount of basic Ti-OH present. In addition, the peaks attributed to species such as Ti^3+^ were not detected on the MAO surface, demonstrating that the substrates were fully oxidized to form stoichimetric TiO_2_, which is consistent with previously published results [20]. The contact angle, which is a representative marker for the hydrophilicity, is strongly correlated with the surface energy of the material. It is known that some material surface properties, such as the surface energy, influence the protein adsorption and the interaction with cells [40]. In the present study, enhanced hydrophilicity was generated on both UV-treated surfaces; however, only the UVC-treated surface showed superhydrophilicity. This is a clear indication of the greater surface energy of the UVC-treated surface compared to the UVA-treated surface, which may be part of the explanation for the increased biological activity. However, the mechanism of the biological activity is not fully understood; protein adsorption and cell attachment processes are complex and controversial [40]–[42]. Some authors have postulated that the surface hydrocarbon content, and not the hydrophilicity, is causally correlated with the protein adsorption and cell attachment, arguing electronic interactions [20]. They propose that when the hydrocarbons are removed by UV treatment, the exposure of Ti^4+^ sites may enhance the attachment of negatively charged proteins and cells to the surface. Interestingly, our results show that the hydrocarbon content was nearly equal after the two different UV treatments, while the Ti 2p peaks for the UVC-treated surface were slightly more intense than for the UVA-treated surface. Thus, further evidence is required to directly link the removal of hydrocarbons and the enhanced affinity of proteins and cells. In another study, superhydrophilicity of a TiO_2_ surface was observed after illumination from a 1000 W high-pressure mercury lamp with a maximum intensity at approximately 365 nm [19], which is considered UVA light. Our present results showed that the contact angle decreased from 65.34±3.47° to 44.64±3.83° after irradiation from a 15 W UVA mercury lamp. This indicates that the enhancement of hydrophilicity is associated with the power of the lamp. Thus, the question of whether more efficient biological activity can be imparted by using higher power UVA light requires further investigation in vitro and in vivo.

The UVC-treated MAO surfaces promote the proliferation of MG-63 cells without sacrificing differentiation. An inverted correlation exists between osteoblastic proliferation and differentiation [43]. In general, enhanced cell differentiation comes at the cost of reduced cell proliferation on a rougher surface [44]. This benefits de novo bone formation around implants at the early stage. However, in accordance with previous studies [20], [22], both the cell proliferation and differentiation was increased by the UVC-treatment of the surface. Possible explanations for this enhancement may be increased intercellular interactions or a larger number of cell signaling pathways, regulated by cellular attachment [24]. Notably, the role that the UVA treatment plays in determining the cell proliferation and differentiation is contested. Although superhydrophilicity has been generated on an acid-etched titanium surface by UVA treatment, almost no effect on the cell proliferation and differentiation was observed [24]. This is in contrast to the enhanced cell proliferation and differentiation on MAO surfaces after illumination from a 1000 W high-pressure mercury lamp [19]. In our study, no proliferation or differentiation enhancement was observed as a result of 15 W UVA mercury lamp irradiation. These differences may result from variations in the surface topography of the titanium and the surface physiochemical character, as well as the power, strength and source of the UV light.

No obvious differences were observed in the surface topography, roughness, elemental composition or the TiO_2_ crystalline character as a result of the ultraviolet treatments. In agreement with previous results, neither UVA nor UVC light affects these physical properties, suggesting that the enhanced biological activity effected by the UV treatment should attributed to chemical changes of the titanium surface.

The basic Ti-OH groups play an important role in enhancing apatite formation. As seen in the XPS spectra, the peak at 532.8 eV, attributed to basic Ti-OH, increased after UV irradiation, especially on the UVC-treated surface. Researchers have reported that the crystal structure [45] and the surface hydroxyl groups [46], as well as the surface roughness of the titanium substrate [47], influence the apatite formation during immersion in SBF. Without considering the role of the crystal structure and surface roughness, the Ti-OH group generated after UV treatment results in a negatively charged MAO coating, which is good for attracting the calcium ions in SBF to the surface. Subsequently, the abundant calcium ions on the surface combine with the phosphate ions in the SBF to form apatite nuclei [48], [49]. In our present study, the increased apatite formation on the UVC-treated surface can be explained by this mechanism. Another study [19] reported a large amount of apatite formation on an MAO surface after UVA treatment; our results showed only slight apatite formation under these conditions. This may be attributed to differences in the power and intensity of the light, as well as the method of irradiation and the frequency at which the SBF is renewed. Variation of these and other parameters may result in better biological activity.

Although histological studies mercury lamp of the bone formation process leading up to osseointegration have been widely researched [13], [14], the mechanism of osseointegration is complex and requires further investigation. Implant surface treatment is one of the determining factors for bone formation directly on the implant surface. From the original TPS (titanium plasma spray) to SLActive, the surface treatments of Straumann implants have experienced a radical transition from physical to chemical, suggesting chemical treatment to be the future trend in dental implant surface treatment. UV light-mediated photofunctionalization of TiO_2_ can induce chemical changes of the titanium surface without sacrificing the excellent surface morphology. The enhanced biological effects imparted by UV treatment have been confirmed in many previous studies; therefore, this technique may be a novel titanium surface treatment for future applications.

### Conclusions

Our findings suggest that the irradiation of an MAO surface with UVC light remarkably enhances the bioactivity of the surface, more so than irradiation with UVA light. The UVC-treated MAO surface promoted biological interaction with proteins and MG-63 cells in the early stage, e.g., protein adsorption, cell attachment and spreading, as well as subsequent cellular proliferation and differentiation. Apatite formation was also increased after UVC irradiation; this effect is again much larger than that seen with UVA irradiation. To test the underlying mechanisms of the two different UV-photofunctionalization methods of titanium, our study examined the hypothesis that different chemical changes occurred on the UVA and UVC-treated MAO surfaces. Although both UVA and UVC irradiation reduced the amount of hydrocarbons on the surface and formed basic Ti-OH groups, the extent of this functionalization varied between the two methods. The presence of more Ti-OH groups may be the reason that the UVC-treated MAO surface exhibits superior bioactivity to the UVA-treated surface.
